# Initial Renal Function (eGFR) Is a Prognostic Marker of Severe Acute Pancreatitis: A Cohort-Analysis of 1,224 Prospectively Collected Cases

**DOI:** 10.3389/fmed.2021.671917

**Published:** 2021-08-18

**Authors:** Pál Tod, Nelli Farkas, Dávid Németh, Gábor Szénási, Áron Vincze, Roland Hágendorn, László Czakó, Dóra Illés, Ferenc Izbéki, Veronika Dunás-Varga, Mária Papp, József Hamvas, Márta Varga, Katalin Gombos, Tamás Nagy, Zsolt Márton, Nándor Faluhelyi, Imola Török, Ali Tüzün Ince, Shamil Galeev, Péter Jenő Hegyi, Andrea Szentesi, Andrea Párniczky, Zsolt Szakács, Péter Hegyi, Péter Hamar

**Affiliations:** ^1^Szentágothai Research Centre, Medical School, Institute for Translational Medicine, University of Pécs, Pécs, Hungary; ^2^School of Medicine, Institute of Translational Medicine, Semmelweis University, Budapest, Hungary; ^3^Division of Gastroenterology, First Department of Medicine, Medical School, University of Pécs, Pécs, Hungary; ^4^First Department of Medicine, Medical School, University of Pécs, Pécs, Hungary; ^5^Department of Medicine, University of Szeged, Szeged, Hungary; ^6^Szent György University Teaching Hospital of Fejér County, Székesfehérvár, Hungary; ^7^Division of Gastroenterology, Department of Internal Medicine, University of Debrecen, Debrecen, Hungary; ^8^Peterfy Hospital, Budapest, Hungary; ^9^Dr. Réthy Pál Hospital, Békéscsaba, Hungary; ^10^Department of Laboratory Medicine, Medical School, University of Pécs, Pécs, Hungary; ^11^Department of Radiology, Medical School, University of Pécs, Pécs, Hungary; ^12^County Emergency Clinical Hospital - Gastroenterology and University of Medicine, Pharmacy, Sciences and Technology, Târgu Mureṣ, Romania; ^13^School of Medicine, Hospital of Bezmialem Vakif University, Istanbul, Turkey; ^14^Saint Luke Clinical Hospital, St. Petersburg, Russia; ^15^Department of Medicine, Centre for Translational Medicine, University of Szeged, Szeged, Hungary; ^16^Heim Pál National Pediatric Institute, Budapest, Hungary; ^17^Centre for Translational Medicine, Medical School, Semmelweis University, Budapest, Hungary; ^18^Division of Pancreatic Diseases, Heart and Vascular Center, Medical School, Semmelweis University, Budapest, Hungary

**Keywords:** AP severity and mortality, renal dysfunction, CKD-EPI, EGFR, human retrospective cohort

## Abstract

**Background:** Acute pancreatitis (AP) is a life-threatening disease. We aimed to explore the prognostic relevance of renal function based on estimated glomerular filtration rate (eGFR).

**Methods:** A prospective registry of AP patients was established by the Hungarian Pancreatic Study Group. Data of 1,224 consecutive patients were collected between 2012 and 2017. Patients were divided into 3 groups according to their eGFR measured within 24 h of hospitalization: *normal* renal function: >90 mL/min, *mild* to moderate renal functional *impairment*: 30–90 mL/min and *severe* renal *dysfunction*: <30 mL/min. Associations of eGFR with outcome (survival, length of hospitalization, AP severity, blood glucose), inflammatory markers (erythrocyte sedimentation rate, white blood cell count), anemia and organ failure (heart, kidney, liver) were analyzed.

**Results:** Death, longer hospitalization and severe AP, but not the cause of AP, were significantly associated with lower eGFR. The inflammatory markers (CRP, WBC count) but not anemia (Hb, Htk) were closely associated with severe renal dysfunction. Renal function was associated with heart and renal failure but not with other complications of AP such as respiratory failure, local pancreatic complications, diabetes or peptic ulcer. eGFR was not associated with liver damage (ALAT, γ-GT) or liver function (serum bilirubin) although biliary complications, alcohol and metabolic syndrome were the most common etiologies of AP.

**Conclusions:** Our study suggests a useful prognostic value of *initial* eGFR in AP patients. Even mild eGFR reduction predicted mortality, severity of AP and the length of hospitalization. Thus, precise evaluation of renal function should be considered for assessing AP severity and outcome.

## Introduction

Acute pancreatitis (AP) is an acute necroinflammation of the pancreas. AP is a leading cause of hospitalization for gastrointestinal disorders (1). Although AP is often mild, the severe form (SAP) develops in 15–20% of patients and has up to 40% mortality (2). AP is diagnosed if at least two of the following three criteria are fulfilled: (1) clinical (abdominal pain consistent with acute pancreatitis), (2) laboratory (serum lipase or amylase >3-fold higher than the upper limit of normal), (3) imaging (characteristic findings of AP by contrast-enhanced computed tomography (CECT), magnetic resonance imaging (MRI) or transabdominal ultrasonography) (based on the International Association of Pancreatology and the American Pancreatic Association IAP/APA guidelines) (3). The incidence of AP ranges from 4.6 to 100 per 100,000 persons in Europe (4), and from 13 to 45 per 100,000 persons in the USA (5). The overall mortality ranges from 2 to 35% depending on whether organ failure or necrotizing pancreatitis occurs (6). The Hungarian Pancreatic Study Group (HPSG) was established in 2011. It developed a prospective electronic registry (https://tm-centre.org/en/registries/acute-pancreatitis-registry/) and collected data of 1,224 patients which served as a basis for this study.

The risk of developing AP is multifactorial. Among the most common causes are age (5) and lifestyle factors such as obesity and alcohol consumption (7). In a previous study, performed on the same cohort, we demonstrated that comorbidities predict mortality and severity of AP (8). Although early severity assessment is crucial for prognosis of outcome, there is no “gold standard” prognostic score for AP (9). Several prognostic systems are based solely on the evaluation of pancreatic necrosis and organ failure [e.g., Atlanta classification, Determinant based classification (DBC), Amylase and BMI (CAB) or the contrast-enhanced Computer Tomography base Severity Index (CTSI)]. Some score systems include other variables (PANC3: Htk, BMI, chest X-ray), CRP: inflammation (C-reactive protein). On the other hand, PANC4 is already extended with the inclusion of blood urea nitrogen (BUN). Some scoring systems include serum creatinine, eGFR or BUN [Revised Atlanta classification (RAC), Ranson's criteria, bedside index of severity in AP (BISAP), Acute Physiology and Chronic Health Examination II (APACHEII), Glasgow score, Harmless AP (10), Charlson Comorbidity Index (CCI)] (9). Thus, several presently used formulas do not include renal function and those which incorporate the most common comorbidities, and thus include renal function to predict AP prognosis, include it only as a simple score without analyzing the severity of renal functional *impairment*.

AP can be associated with circulatory shock-induced organ failure, including renal damage, potentially leading to acute kidney injury (AKI). The exact mechanism of AP-induced AKI (or renal failure) is yet to be fully understood, but besides hypotension the common causes can be systemic inflammation with cytokine production and free radical species, leading to renal parenchymal damage and hypoxemia (11, 12). Tissue damage and hypoxia stimulate further inflammation and oxidative stress leading to a vicious cycle. Although, organ damage or failure is well-known to severely impact the outcome of AP, the effects of early renal *impairment* on AP outcome have not been studied in depth before.

The aim of this study was to investigate possible associations between AP outcome and the clinical gold standard biomarker of renal function, the chronic kidney disease epidemiology (CKD-EPI) formula-based estimated glomerular filtration rate (eGFR) measured within the first 24 h after hospitalization.Article types.

## Methods

Patients with AP were enrolled to the multicenter-based electronic database of the Hungarian Pancreatic Study Group (HPSG) – acute pancreatitis Registry. The data were collected from 2012 to 2017. AP diagnosis was established according to the IAP/APA guidelines (3). The following symptoms were considered in each patient: abdominal pain (clinical symptom), pancreatic enzyme elevation at least three times above the upper limit (laboratory data) and morphological changes (imaging). Patients were included in the registry if at least 2 out of the 3 criteria were fulfilled. In order to gain a more detailed insight into the possible associations and predictive role of estimated glomerular filtration rate (eGFR), the present study is based on data of all patients included in the registry, irrespective of the pancreatic enzyme elevation at the time of eGFR test.

Eighty-six parameters were registered altogether. Seventy-seven percentage of the requested data were provided by the investigators. The remaining missing data were either not measured or not investigated. For further details, see Parniczky et al. (13).

In this study 1,224 patients were included. Serum creatinine based eGFR was measured within 24 h following hospitalization. Patients were divided into 3 groups based on their eGFR values derived from their respective serum creatinine values and calculated according to the chronic kidney disease epidemiology (CKD-EPI) formula, by gender:

$$\begin{array}{l} CKD-EPI=141\\ \times {\frac{minimum\;serum\;creatinine}{0.7}}^{male:\;-0.411,\;female:-0.329\;}\\ \times {\frac{maximum\;serum\;creatinine}{0.7}}^{-1.209}\\ \times 0.993^{age\;of\;the\;patient}\times 1.018\;if\;female \end{array}$$

The correction factor for black race was not necessary in this registry. Creatinine was measured by the modified Jaffe reaction on a Hitachi 911 automatic analyzer (Roche Diagnostics, Mannheim, Germany).

The 3 groups were the following: patients with eGFR <30 mL/min (*n* = 44), eGFR: 30–90 mL/min (*n* = 630) and eGFR >90 mL/min (*n* = 550). A matched control group was selected from patients with eGFR >90 mL/min (*n* = 39) to match by age (± 5 years) and sex with the eGFR <30 mL/min group. Most evaluations were performed including the matched control group eGFR >90 mL/min (*n* = 39) and the non-matched patients with eGFR >90 mL/min (*n* = 511) were not included in the study.

The severity of AP was evaluated based on the revised Atlanta classification (14). Mild AP was characterized by the lack of organ failure, lack of local or systemic complications and if resolved within the first week. Moderate AP was characterized by transient organ failure, local complications or exacerbation of any co-morbid disease. Severe AP was characterized by persistent (>48 h) failure of one or more organs.

Analyzed parameters were defined as follows: Organ failure was defined according to the modified Marshal scoring system (15). New onset diabetes was defined as no diabetes (lack of documented or reported diabetes history, lack of antidiabetic medication and HbA1C in reference range) at the time of hospitalization due to AP, but diabetes developed as a consequence of the AP episode and the patient was released with appropriate antidiabetic medication. Comorbidities, such as peptic ulcer, myocardial infarction and vascular disease were recorded if the patient reported it or the patient's medical documentation included it. Symptoms and complaints (weight loss, loss of appetite, nausea, vomiting) as well as life-style attributes (smoking, alcohol consumption) were recorded based on the patients' reports.

### Ethical Approval

The multicenter-based electronic database of the HPSG – AP Registry was approved by the Scientific and Research Ethics Committee of the Medical Research Council (22254-1/2012/EKU). All patients were informed about the data collection and signed the informed consent forms.

### Statistics

The results are expressed as median ± interquartile range (IQR). Data was analyzed after logarithmic transformation in case of significant inhomogeneity of variances indicated by Bartlett's test. One-way ANOVA was used for multiple comparisons followed by Tukey's or Bonferroni *post-hoc* test. Kruskal-Wallis test was performed if normal distribution was not met. Unpaired *t*-test with Welch's correction was used to analyze two groups and chi-square test was used to analyze patient frequencies.

## Results

### Better Initial Renal Function Was Associated With Better Outcome of Acute Pancreatitis

**Figure 1:**
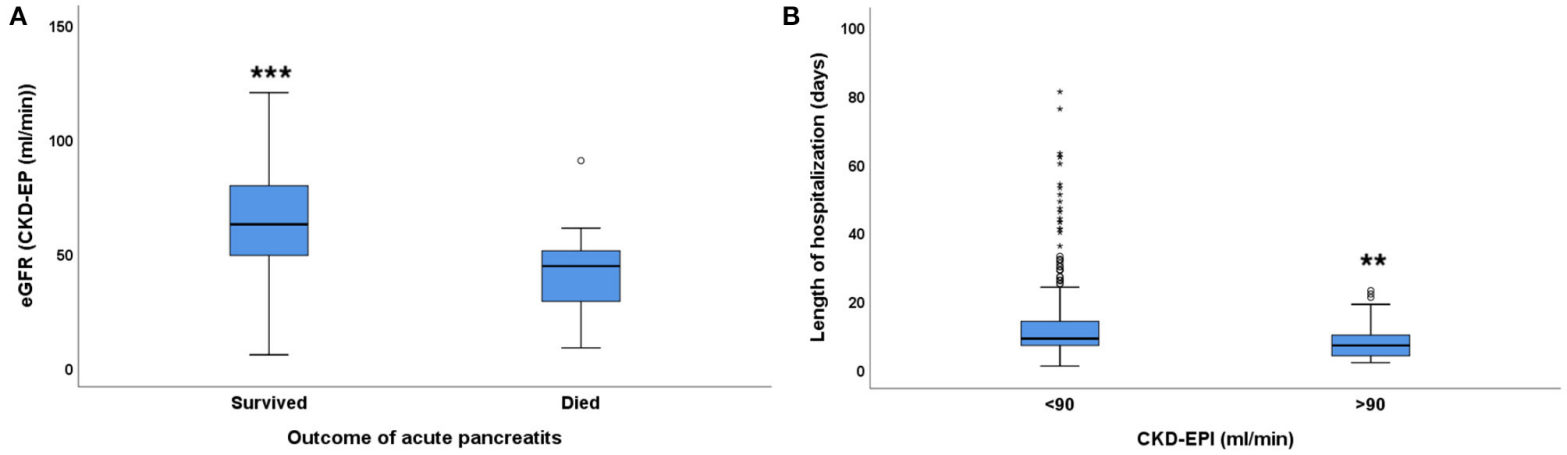
The relationship between eGFR and acute pancreatitis outcome. **(A)** mortality, **(B)** length of hospitalization in days; median ± IQR, unpaired *t*-test with Welch correction, **p = 0.001, ***p < 0.001. Small circles and stars represent outlier data.

In the present study the average eGFR (24 h within hospitalization) was significantly lower in patients who died, compared to patients who survived (Figure 1A). Mortality was higher in patients with impaired renal function: 21/257 patients (8.17%) died if eGFR was <60 mL/min, whereas only 10/279 (3.58%) patients died if eGFR was >60 mL/min. Mild to moderate reduction of renal function (eGFR <90 mL/min) was already associated with significantly longer hospitalization (12.13 ± 0.45 days vs. 8.56 ± 0.93 days) (Figure 1B).

**Figure 2:**
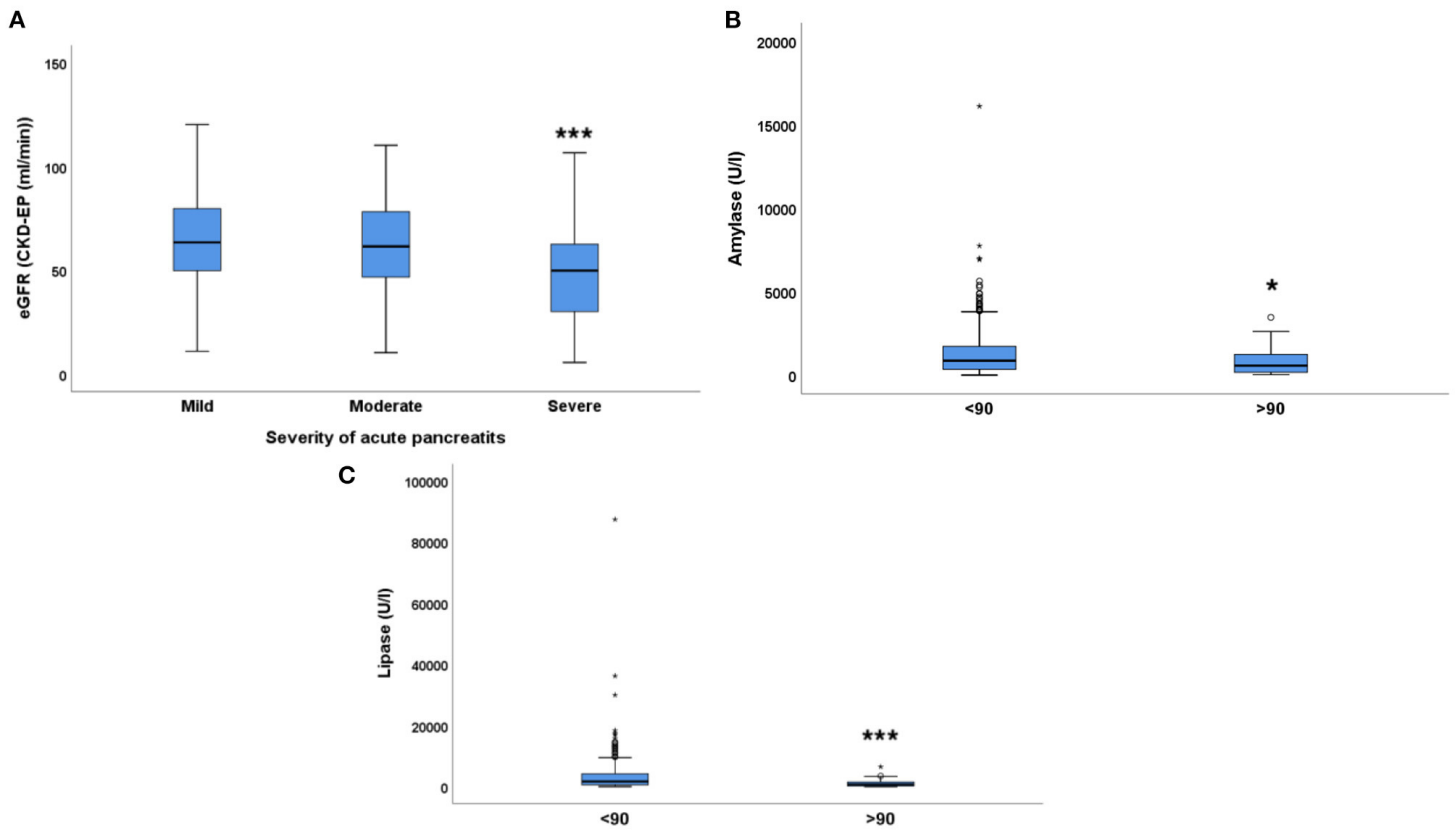
The relationship between eGFR and acute pancreatitis severity. **(A)** severity of acute pancreatitis, **(B)** Amylase (U/L), **(C)** Lipase (U/L); median ± IQR, **(A)** one-way ANOVA with Bonferroni *post-hoc* test, ***p = 0.002 (severe vs. moderate); *p* < 0.001 (severe vs. mild). **(B,C)** unpaired *t*-test with Welch correction. *p < 0.01, ***p < 0.001. Small circles and stars represent outlier data.

There was a strong association between the eGFR and the severity of acute pancreatitis (AP), based on the revised Atlanta classification: eGFR was significantly lower (48.53 ± 3.84) in patients with severe AP (*n* = 47). However, mild to moderate renal functional *impairment* was not associated with AP severity, as eGFR was similar in patients with mild (63.64 ± 1.06 mL/min, *n* = 365) or moderate (61.18 ± 1.93 mL/min, *n* = 124) AP (Figure 2A). Exocrine pancreatic enzymes in the blood were elevated in all patients with AP suggesting pancreatic cell damage. Amylase (reference range: 40–140 U/L) and lipase (reference range: 0–50 U/L) values were lower (amylase: 848.8 ± 151.6 U/L, lipase: 1,298 ± 343.3 U/L) in patients with eGFR >90 mL/min than in patients with eGFR <90 mL/min (Figures 2B,C). Already a mild to moderate reduction of eGFR (30–90 mL/min) was accompanied by significantly higher amylase and lipase activities (amylase: 1,265.1 ± 444.4 U/L, lipase: 3,535.9 ± 2,199.8 U/L, *p* < 0.05). However, eGFR <30 was not accompanied by further elevation of the exocrine pancreatic enzyme activity in the blood (amylase: 1,099.7 ± 1,179.6 U/L, lipase: 2,226 ± 3,134.4 U/L).

Interestingly, subfebrility or fever was also associated with renal function. Significantly higher portion of patients with eGFR<30 mL/min (33%) had subfebrility or fever than in the patient groups with mild to moderately impaired (eGFR: 30–90: 16%) or normal renal function (eGFR >90 mL/min: 17%) (*p* = 0.022).

**Figure 3:**
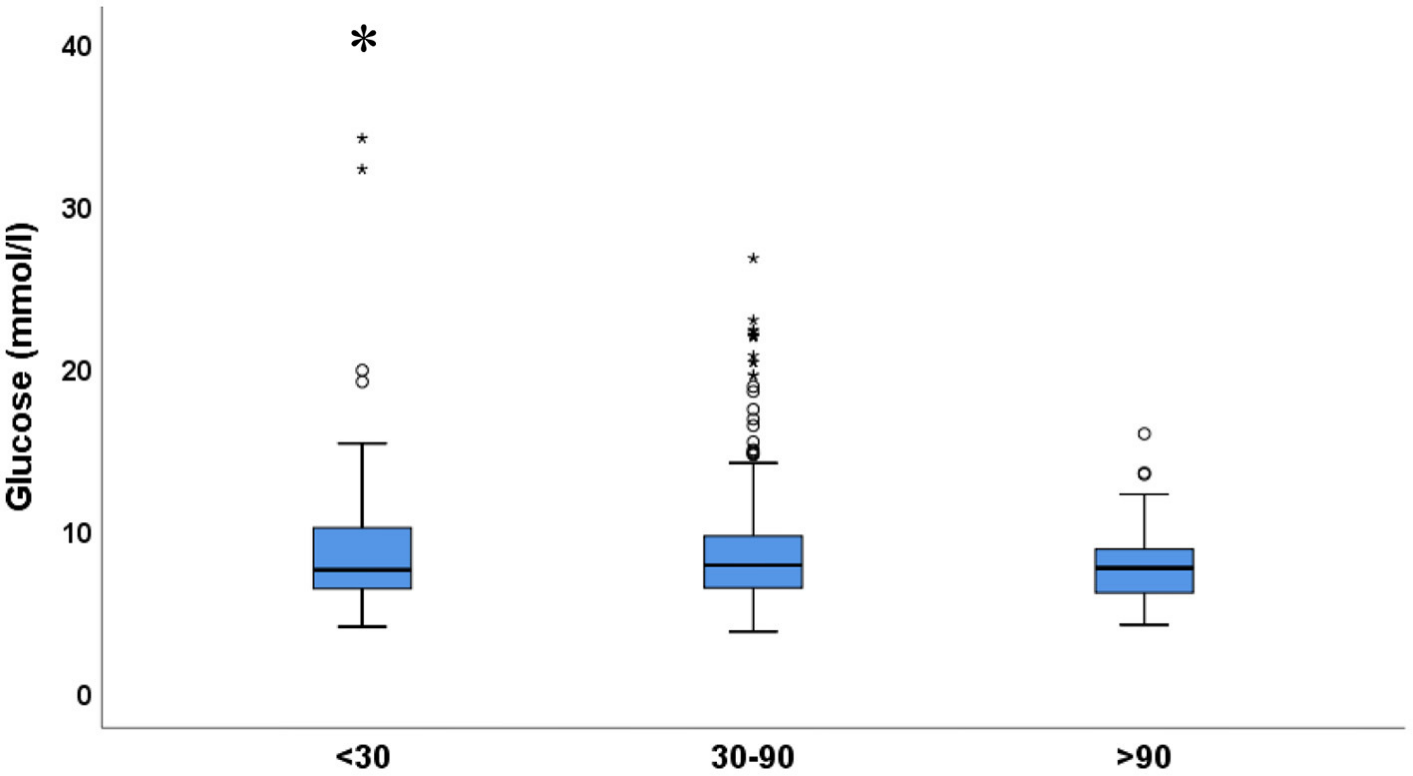
Blood glucose in the 3 eGFR groups. Values are expressed as median ± IQR. One-way ANOVA with Tukey's *post-hoc* test; *p < 0.05. Small circles and stars represent outlier data.

On the other hand, renal function was not associated with the severity of endocrine *dysfunction* of the pancreas, i.e., diabetes. Blood glucose as well as the incidence of new onset diabetes was similar in all eGFR groups. There was a tendency of higher blood glucose values in some patients with low eGFR but blood glucose was within the reference range in most patients with normal renal function (eGFR >90 mL/min), however, this association was not statistically significant (Figure 3).

### The Cause of Acute Pancreatitis Was Not Associated With eGFR

Regarding the etiology of acute pancreatitis, AP was caused by biliary complications (54.3%) in most of the cases, followed by idiopathic (24.1%) and alcohol-induced AP (12.4%). Further causes are listed in Table 1. There was no significant association between eGFR and the cause of AP (biliary: *p* = 0.1941, idiopathic: *p* = 0.7141, alcohol-induced: *p* = 0.6114 and metabolic syndrome associated: *p* = 0.1153) (Table 2). Forty-three patients (8.02%) had multiple etiologies, mainly biliary and lipid metabolism disorders. In our cohort, possible etiologies such as cystic fibrosis, genetic defects, gluten sensitive enteropathy and virus infection were not found in any of the patients.

**Table 1:** Causes of acute pancreatitis.

**Etiology**	**Number of patients**
Biliary	291
Idiopathic	129
Alcohol-induced	74
Other	62
Metabolic syndrome (MS)	19
Post ERCP	7
Congenital malformation	1
Drug induced	1
Trauma	10
No data	58

**Table 2:** The association between major causes of AP and eGFR status.

**Cause of AP**	**Total**	** <30**	**30–90**	**>90**
Biliary-complications	291	20 (6.9%)	255 (87.6%)	16 (5.5%)
Idiopathic	129	13 (10%)	108 (83.7%)	8 (6.2%)
Alcohol-induced	74	6 (8.1%)	65 (87.8%)	3 (4%)
MS-induced	19	4 (21%)	14 (73.7%)	1 (2.3%)

*Patient number (percentage). MS, metabolic syndrome*.

### The Inflammatory Markers and Hyperlipidemia but Not Anemia Were Closely Associated With Severe Renal Dysfunction

**Figure 4:**
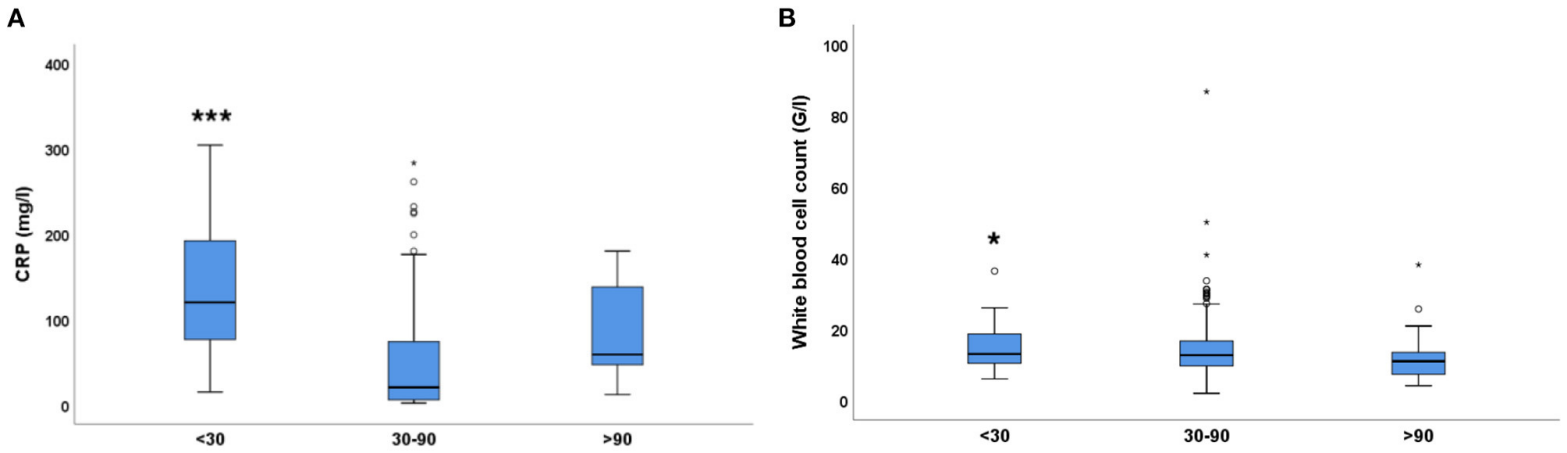
Inflammatory parameters (CRP, WBC count) in the 3 eGFR groups. **(A)** C-reactive protein (CRP, mg/L), One-way ANOVA with Tukey's *post-hoc* test; ***p < 0.001. **(B)** White blood cell count (10^9^/L), Independent samples Kruskal Wallis test; *p = 0.05 (<30 vs. >90). Small circles and stars represent outlier data.

C-reactive protein (CRP), a marker of inflammation was elevated in most patients (reference range: <5 mg/L). Average CRP was similarly elevated in patients with normal renal function (eGFR > 90 mL/min, CRP: 51.35 ± 10.64 mg/L) and in patients with mild to moderate eGFR reduction (30–90 mL/min, CRP: 53.39 ± 3.55 mg/L). However, CRP was more elevated in patients with *severe renal dysfunction* (eGFR <30 mL/min) (CRP: 132.7 ± 18.47 mg/L) that in patients in the other two groups (Figure 4A).

Another inflammatory marker, white blood cell (WBC) count (reference range: 4–11 × 10^9^/L) was close to reference range only in patients with eGFR >90 mL/min (WBC: 11.75 ± 1.1 G/L) (Figure 4B). There was a small trend for WBC count elevation (13.6 ± 0.3 × 10^9^/L, ns) in patients with mild to moderately reduced renal function (eGFR: 30–90 mL/min). However, severe renal dysfunction (eGFR<30 mL/min) was accompanied by a significant increase in WBC count (14.8 ± 0.9 × 10^9^/L). Taken together, CRP and WBC count were significantly higher in patients with severe renal impairment, suggesting an association between these parameters.

Hyperlipidemia was more common in patients with severe renal functional impairment. In 37% of patients with eGFR <30 mL/min had hyperlipidemia whereas in patients with eGFR >30 mL/min only 19% had hyperlipidemia (*p* = 0.019).

Regarding the anemia status of the patients, hematocrit (htc) values were similar, irrespective of renal function. However, a tendency of a gradual decrease in hemoglobin concentrations was observed in parallel with the decline in eGFR values (Table 3).

**Table 3:** The relationship between anemia status and renal function.

**Renal function (eGFR) (mL/min)**	**Hemoglobin (U/L)**	**Hematocrit (%)**
>90	150.8 ± 6.9	40.8 ± 1.1
30–90	140.3 ± 1.2	41.3 ± 0.3
<30	134.5 ± 3.9	40.0 ± 1.1

*Values are expressed as mean ± SEM. One-way ANOVA with Tukey's post-hoc test*.

### Renal Function Was Associated With Heart and Renal Failure and Vascular Complications but Not With Complications of Acute Pancreatitis

Concerning other complications of AP eGFR <90 mL/min was strongly associated with heart and renal failure episodes, but not with respiratory failure. None of the patients with eGFR >90 mL/min had heart or renal failure. Heart or renal failure was rare in patients with eGFR: 30–90 mL/min but was relatively common in patients with eGFR<30 mL/min.

**Figure 5:**
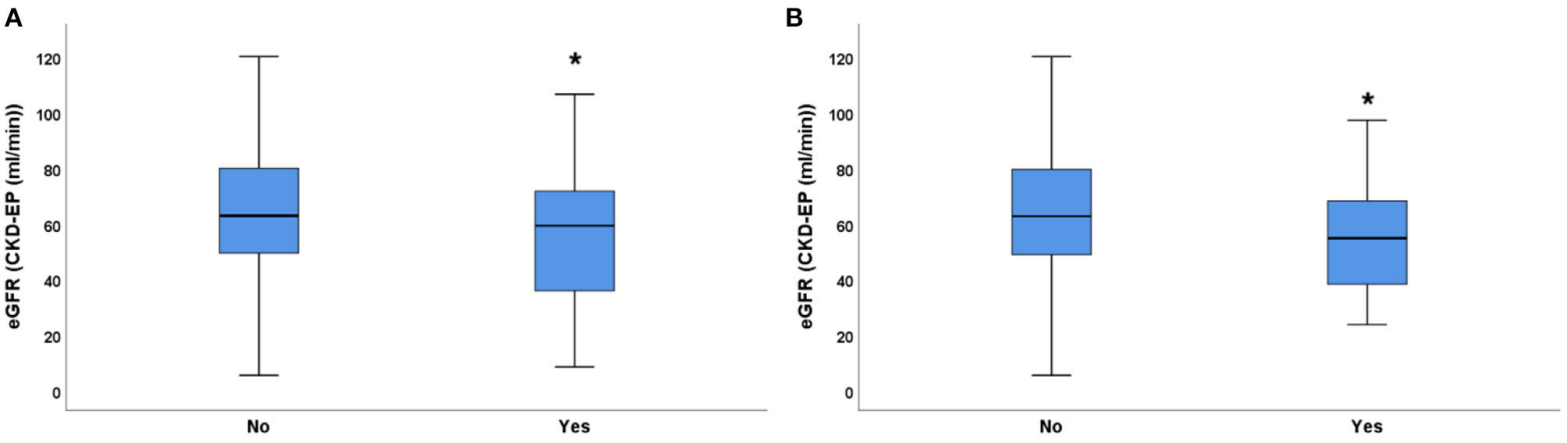
EGFR values in patients with or without peripheral vascular disease and myocardial infarction. **(A)** peripheral vascular disease (verified atherosclerosis) **(B)** myocardial infarction. Unpaired *t*-test with Welch's correction. *p < 0.05.

The presence and complications of peripheral vascular disease (PVD) were also associated with eGFR. Patients with PVD (verified atherosclerosis) had significantly lower eGFR (Figure 5A). Furthermore, PVD was significantly associated with eGFR (Table 4). Patients with acute myocardial infarction (AMI) also had significantly lower eGFR (Figure 5B). Although, AMI was least common in patients with eGFR >90 mL/min and most common with eGFR <30 mL/min, this association was not statistically significant.

**Table 4:** Complications of (A) acute pancreatitis, (B) vascular disease.

**Complication**	** <30 mL/min**	**30–90 mL/min**	**>90 mL/min**	***P* =**
**(A)**				
Heart failure	5 (11.6%)	10 (2.2%)	0 (0%)	0.001
Respiratory failure	5 (11.6%)	30 (6.6%)	4 (11.4%)	ns
Local pancreatic complication	20 (45.4%)	150 (33%)	12 (33.3%)	ns
Other local complication	3 (6.8%)	12 (2.6%)	1 (2.8%)	ns
New onset diabetes	4 (9.1%)	20 (4.4%)	2 (5.6%)	ns
Peptic ulcer	1 (2.3%)	50 (12.0%)	4 (13.3%)	ns
**(B)**				
Peripheral vascular disease	11 (30%)	58 (13%)	0 (0%)	0.01
Myocardial infarction	5 (13.5%)	30 (7.2%)	2 (6.7%)	ns

*Patient number (percentage). Chi-Square test*.

The incidence of local pancreatic complication or other local complications, diabetes as complication of AP and peptic ulcer was similar in all eGFR groups (Table 4). Thus, renal function was an independent prognostic indicator selectively for cardiovascular and renal complications of acute pancreatitis.

### There Was No Significant Association of eGFR With Liver Damage or Liver Function

As biliary complications, alcohol consumption and metabolic syndrome were the most common etiologies of AP, we evaluated the association of liver and renal functions with AP. Although, significant associations were not found, liver enzymes such as ALAT (reference range: <40 U/L) and γ-GT (reference range: 9–48 U/L) were higher in patients with lower eGFR suggesting that functional *impairment* of the kidney and liver damage were parallel. There was no association between total bilirubin (reference range: <20 μmol/L) and eGFR (Table 5).

**Table 5:** Association between liver- and renal function.

**Renal function (egfr) (ml/min)**	**Total bilirubin (μmol/L)**	**ALAT (U/L)**	**γ-GT (U/L)**
>90	49.02 ± 8	95.87 ± 19.8	289.8 ± 70.8
30–90	39.8 ± 2	147.1 ± 11.4	351.7 ± 23.4
<30	46.18 ± 10.3	168.5 ± 41.6	360.8 ± 85.2
p:	ns	ns	Ns

*Values are expressed as mean ± SD. One-way ANOVA with Tukey's post-hoc test*.

### eGFR Was Not Associated With Life-Style Attributes or Gastrointestinal Symptoms

Current alcohol consumption and smoking habits as confessed by the patient were similar in all eGFR groups (data not shown). Furthermore, gastrointestinal symptoms of AP such as weight loss, appetite, nausea and vomiting were not associated with eGFR either (data not shown). Although appetite loss was most common in patients with eGFR<30 mL/min, and vomiting was less common in patients with eGFR >90 mL/min these differences were not significant.

## Discussion

This is the first paper describing the associations between AP and biomarkers of renal function, assessed by creatinine-based eGFR within 24 h of hospitalization, in patients enrolled in our prospective AP registry, a large cohort with high quality data. Renal *dysfunction* is regarded as a late complication in AP. However, according to our analysis, even early and mild to moderate renal functional *impairment* has a prognostic value for both severity and outcome.

Better initial renal function was associated with better outcome, such as longer survival and shorter length of hospitalization. In line with this observation, eGFR strongly correlated with severity of AP and blood levels of exocrine pancreatic enzyme levels. Furthermore, eGFR was significantly associated with subfebrility/fever, systemic inflammatory parameters (CRP, WBC count) hyperlipidemia and vascular complications (atherosclerosis, myocardial infarction, heart- and renal-failure). Observational studies, such as the current registry analysis do not enable to establish causative relationships (16). Thus, we can only conclude that survival was better if initial eGFR was >60 mL/min, length of hospitalization increased if eGFR was <90 mL/min, severe pancreatitis was associated with eGFR<30 mL/min and blood glucose was elevated if eGFR was <30 mL/min. Blood amylase and lipase activities were significantly elevated if initial renal function was slightly impaired (eGFR <90 mL/min). Several investigated parameters are either comorbidities or complications, such as peripheral vascular disease and consequent myocardial infarctions, which were more common in low eGFR patients. Although, causative relationships are not proven, preexisting atherosclerosis is a known risk factor for inferior outcome of AP and is associated with low eGFR. However, the observed association raises the possibility that complications like myocardial infarction are more likely to occur if renal function is impaired. The lack of association between eGFR reductions and increases in pancreatic damage markers (amylase, lipase) is best explained by the rapid decline of pancreatic enzymes in the blood after pancreatitis, often resulting in reference range values already at the time of diagnosis.

In our registry, AP severity was defined based on the Atlanta scoring system, including renal failure (eGFR <15 ml/min) and this could influence the association between severity and eGFR. However, renal failure was 1 of 4 complications (Heart-, Respiratory- Renal- and Systemic organ- failure) and further 6 pancreas related complications (Local pancreatic-, Fluid collection, Pseudocyst, Necrosis, Diabetes, and other local complications) were included in the severity score. Furthermore, only 13 patients had an eGFR <15. Thus, the detected associations between the initial eGFR and severity is not likely to be due to the inclusion of renal failure in the definition of severity. Association of renal function and pancreatitis outcome has been reported before: eGFR <90 mL/min was a predictor of pancreatic necrosis (17) and a BUN increase >1.8 mmol/L had a high predictive value as a single parameter for severe AP (18). It has been reported recently that within the first week of the disease, acute kidney injury (AKI) is among the most common causes of mortality in AP, termed as AP-renal syndrome (19). A recent study suggested high serum creatinine to be a marker of pancreatic necrosis (20). Although most severity scores include eGFR, a detailed renal functional evaluation is still not regarded as an important prognostic parameter for AP severity and outcome (9).

In such a large, multicenter cohort demographic distribution may have a significant effect. However, as most centers included in our registry were central-eastern European with only 2 patients from Japan (Supplementary Figure 1) the cohort was homogenously Caucasian. Average age was 65 spanning from 15 to 95 and a bit over the half of the patients were males (Supplementary Figure 3). The leading cause of AP was biliary complications (54.29%). Alcohol induced AP and idiopathic forms (together: 36.49%) were less common than biliary complications. These observations are in line with literature data. In several studies, gallstones were the most frequent etiology of AP followed by alcohol-induced AP (21–23). Liver function (as assessed by serum bilirubin) did not correlate with eGFR, suggesting that impaired liver function did not contribute to the more severe outcome of the patients with eGFR <30 ml/min. As – similarly to the kidney – the liver is affected in AP by hypoxia due to centralization of the circulating blood and by toxicity of circulating exocrine enzymes, we have observed a non-significant association of ALAT and γ-GT with eGFR suggesting that functional *impairment* of the kidney and liver damage were parallel.

We detected no association between the cause of AP and initial eGFR in this registry. One report suggests that biliary pancreatitis could lead to renal insufficiency (24). Our analysis did not support this observation, probably due to a relatively low number of patients with renal *impairment* in our cohort.

In our study, severe initial renal *dysfunction* (eGFR < 30 ml/min) was associated with significant elevation of systemic inflammatory markers, such as CRP and white blood cell count. Similar observations have been reported before describing the role of pancreatic enzyme release due to autodigestion of acinar cells of the pancreas showing that AP can trigger AKI by various stimuli such as inflammation, vasoconstriction and hypoxia (11, 12). Moreover, as amylase is partly excreted by the kidneys serum amylase concentration can be even more increased in patients with initial renal dysfunction (25). However, the importance of amylase plasma concentration is limited for establishing the diagnosis of pancreatitis (26). Erythrocyte sedimentation rate (ESR), another common inflammatory marker was not assessed in the present study, as only a minority of patients had ESR data in our cohort.

Although one would expect an association between the anemia status (Hb, Hct) and reduced eGFR, especially as eGFR was strongly associated with the severity of acute pancreatitis, our data did not support any association between eGFR and Hct. A slight correlation between eGFR and Hb supports that Hb is a more accurate parameter to describe anemia status than Hct. A possible conclusion from the observation that eGFR was not strongly associated with anemia is that AP influenced eGFR and not the other way around, as acute kidney injury is expected to increase plasma erythropoietin concentration. As the patients were hospitalized after the diagnosis of AP, we have no data about their eGFR before the onset of the disease.

Limitation of the study is that we have no creatinine values before the onset of the AP episode. Although renal *impairment* was probably a consequence of AP in most cases, as peripheral vascular disease was seldom present in the cohort, we were not able to differentiate between preexisting renal disease or acute decline of renal function due to AP. Thus, the causative role of renal *impairment* could not be investigated. However, registry analysis is not suitable for establishing causative linkages, anyway. Thus, despite this limitation, our presented data are conclusive about the predictive value of initial eGFR irrespective whether the cause of low eGFR is a preexisting renal *impairment* or is caused by AP.

## Conclusion

eGFR is a good predictor of the prognosis of acute pancreatitis and should be regarded as an important prognostic index independent of other complications of acute pancreatitis. As even mild to moderate eGFR reduction predicted survival, severity, comorbidities and outcome, a detailed evaluation of renal function is suggested for improving the prediction of acute pancreatitis prognosis.

## Data Availability Statement

The raw data supporting the conclusions of this article will be made available by the authors, without undue reservation.

## Author Contributions

NFar, GS, and PHa: conceptualization. PT, NFar, DN, GS, and PHa: data curation. NFar, DN, GS, ZS, PHe, and PHa: formal analysis. PHe: funding acquisition. ÁV, RH, LC, DI, FI, VD-V, MP, JH, MV, KG, TN, ZM, NFal, IT, AI, SG, PJH, AS, AP, and ZS: investigation. TP, NFar, DN, GS, ZS, PHe, and PHa: methodology. PJH, AS, AP, ZS, and PHe: project administration. PHe: resources. PHa and PHe: supervision. NFar, AS, PHa, and PHe: validation. PT, GS, and PHa: writing–original draft. PHa: writing–review & editing. All authors contributed to the article and approved the submitted version.

## Conflict of Interest

The authors declare that the research was conducted in the absence of any commercial or financial relationships that could be construed as a potential conflict of interest.

## Publisher's Note

All claims expressed in this article are solely those of the authors and do not necessarily represent those of their affiliated organizations, or those of the publisher, the editors and the reviewers. Any product that may be evaluated in this article, or claim that may be made by its manufacturer, is not guaranteed or endorsed by the publisher.
