# Supplementary material for: Initial Renal Function (eGFR) Is a Prognostic Marker of Severe Acute Pancreatitis: A Cohort-Analysis of 1,224 Prospectively Collected Cases
Source: Front Med (Lausanne). 2021 Aug 18;8:671917. doi: 10.3389/fmed.2021.671917 (PMC8416275; doi:10.3389/fmed.2021.671917)
Supplement: Supplementary file 1 [file Data_Sheet_1.pdf]

Supplementary Material

S1 Fig. Distribution of participating centers

Country	Institute	Case no.
Hungary	First Department of Medicine, Medical School, University of Pécs	317
	Department of Medicine, University of Szeged, Szeged,	232
	Szent György University Teaching Hospital of Fejér County, Székesfehérvár,	185
	Bajcsy-Zsilinszky Hospital, Budapest,	100
	Department of Internal Medicine, Division of Gastroenterology, University of Debrecen, Debrecen,	73
	Dr. Réthy Pál Hospital, Békéscsaba,	45
	Pándy Kálmán Hospital of Békés County, Gyula,	25
	Markusovszky University Teaching Hospital, Szombathely,	9
	Borsod-Abaúj-Zemplén County Hospital and University Teaching Hospital, Miskolc,	9
	Bács-Kiskun County Hospital, Kecskemét,	8
	Polyclinic of Hospitaller Brothers of Saint John of God	5
	Dr. Bugyi István Hospital, Szentes,	3
	Department of Surgery, University of Szeged, Szeged,	3
	Healthcare Center of County Csongrád, Makó,	2
	Institute of Surgery, University of Debrecen, Debrecen,	2
	Department of Gastroenterology, Medical Centre, Hungarian Defence Forces, Budapest,	2
	Heim Pál National Pediatric Institute	1
Romania	County Emergency Clinical Hospital - Gastroenterology and University of Medicine, Pharmacy, Sciences and Technology, Targu Mures,	40
Lithuania	Vilnius University Hospital Santaros Clinics, Vilnius,<br>Clinics of Abdominal Surgery, Nephrourology and Gastroenterology, Faculty of Medicine, Vilnius University, Vilnius,	31
Spain	Consorci Sanitari del Garraf, Sant Pere de Ribes, Barcelona,	30
Finland	Helsinki University Hospital and University of Helsinki, Helsinki,	27
Japan	Keio University, Tokyo,	2
Belarus	Gomel Regional Clinical Hospital, Gomel,	8
Ukraine	Bogomolets National Medical University, Kiev,	8
Latvia	Gastroenterology, Hepatology and Nutritional Centre, Pauls Stradins Clinical University Hospital, Riga,	8
Turkey	Hospital of Bezmialem Vakif University, School of Medicine, Istanbul,	20
Russia	Saint Luke Clinical Hospital, St. Petersburg,	18
Czech Republic	Centrum péče o zažívací trakt, Vítkovická nemocnice a.s., Ostrava,	11

	Total number of patients	1224

S2 Fig. Quality of Prospectively collected data

In the original cohort 86 different parameters were collected. In the present study the following 35 parameters were analyzed:

Epidemiology and etiology	Overall	Uploaded	%
1. Age	1224	1224	100%
2. Gender	1224	1224	100%
3. Etiology	1224	1224	100%
4. Days of hospitalization	1224	1224	100%
5. AP Severity (mild/moderate/severe)	1224	1224	100%
6. Mortality	1224	1224	100%
7. Fever	1224	1215	99.3%
Average uploaded data	1224	1223	99.9%
Diagnosis, Anamnestic Data and Symptoms			
Life-style attributes:			
8. smoking	1224	1218	99.5%
9. alcohol consumption	1224	1219	99.6%
10. diabetes	1224	1213	99.1%
Gastrointestinal symptoms:			
11. weight loss	1224	1133	92.6%
12. loss of appetite	1224	865	71%
13. nausea	1224	1167	95.3%
14. vomiting	1224	1186	96.9%
Average uploaded data	1224	1143	93.4%
Laboratory parameters at admission			
15. serum creatinine	1224	1224	100%
16. blood glucose	1224	1145	93.5%
17. pancreatic enzymes (Amylase, Lipase)	1224	775	63.3%
Liver function:			
18. Total bilirubin	1224	1107	90.4%
19. alanin amino transferase (ALAT)	1224	676	55.2%
20. gamma-glutamyl transaminase (γ-GT)	1224	1024	83.7%
Serum lipids			
21. cholesterol	1224	179	14.5%
22. triglycerides	1224	234	19.2%
Inflammatory parameters:			
23. C-reactive protein (CRP)	1224	1130	92.3%
24. erythrocyte sedimentation rate (ESR)	1224	127	10.4%
25. white blood cell count	1224	1208	98.7%
Anemia status:			

26. hemoglobin (Hg)	1224	856	69.9%
27. hematocrit (Htk)	1224	853	69.7%
Average uploaded data	1224	810.6	66.2%
Complications/Comorbidities			
28. Renal failure	1224	1219	99.6%
29. Heart failure	1224	1219	99.6%
30. Respiratory failure	1224	1219	99.6%
31. Local pancreatic complication	1224	1219	99.6%
32. Other local complication	1224	1220	99.7%
33. New onset diabetes	1224	1220	99.7%
34. Peptic ulcer	1224	1028	83.9%
35. peripheral vascular disease	1224	1028	83.9%
36. myocardial infarction	1224	1028	83.9%
Average uploaded data	1224	1015.5	94.4%
Overall	44064	37498	85.1%

eGFR was calculated for each patient based on the recorded gender, age and creatinine data and the CKD-EPI formula.

S3 Fig. Figures of demography and representativeness of the study population

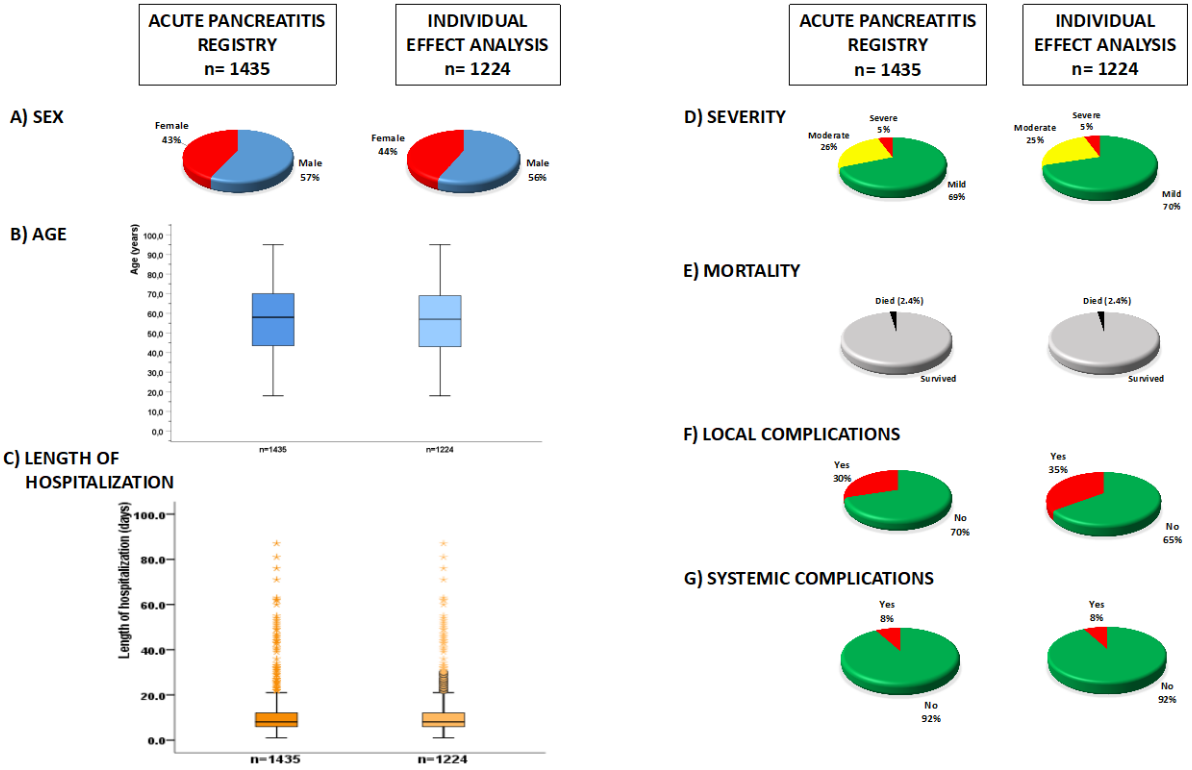

Total AP Registry (n=1435) vs. individual effect analysis (n=1224).
